# Relationship between oral function and life‐space mobility or social networks in community‐dwelling older people: A cross‐sectional study

**DOI:** 10.1002/cre2.381

**Published:** 2020-12-15

**Authors:** Motoyoshi Morishita, Taeka Ikeda, Natsue Saito, Mihoko Sanou, Mayumi Yasuda, Shigeko Takao

**Affiliations:** ^1^ Department of Physical Therapy Kibi International University Takahashi Japan; ^2^ Community General Support Center Asakuchi City Office Asakuchi Japan; ^3^ Department of Health and Welfare Asakuchi City Office Asakuchi Japan; ^4^ Department of Nursing Kibi International University Takahashi Japan

**Keywords:** community‐dwelling older people, life‐space mobility, oral function, social networks

## Abstract

**Objectives:**

This study aimed to clarify the relationship between oral function and life‐space mobility or social networks in order to explore approaches that prevent a decline in oral function.

**Materials and methods:**

A total of 113 community‐dwelling older people (mean age; 75.7 ± 7.3 years) who participated in preventive long‐term care projects aimed at the maintenance or improvement of physical and mental functions were included in this study. The life‐space assessment (LSA) was used to evaluate life‐space mobility, while the Lubben Social Network Scale‐6 (LSNS‐6) was employed to assess social networks. Oral function was measured by maximum voluntary tongue pressure, oral diadochokinesis, the repetitive saliva swallowing test, and lip pressure. Indices of frailty were grip strength and the Kihon Checklist score. Multiple regression analysis was performed to clarify whether the LSA, LSNS‐6, and frailty are associated with oral function.

**Results:**

The results of the Kihon Checklist showed that 63 participants (56%) were subjectively and at least slightly aware of a decline in oral function. LSA (*B* = 0.222, *p* < .001) and grip strength (*B* = 0.266, *p* = .003) associated with maximum voluntary tongue pressure. The goodness of fit of the predictive model was an adjusted *R* ^2^ value of .486. Other oral functions were not associated with any factors and the goodness of fit of the model was poor (Adjusted *R* ^2^ < .1). LSNS‐6 was not associated with any oral function.

**Conclusions:**

Life‐space mobility and grip strength were independent factors associating maximum voluntary tongue pressure, while social networks did not directly association oral function. This study suggests the necessity of a program that approaches both oral and physical functions through guidance for securing a certain amount of daily activity for older people at risk of or exhibiting a decline in maximum voluntary tongue pressure.

## 1 INTRODUCTION

In 2017, one in eight people worldwide was aged 60 years or older. In 2050, older people are projected to account for one in five people globally (United Nations, n.d.). In 2015, the Japanese life expectancy was 80.8 years for men and 87.0 years women and the proportion of people aged 65 years or older was 26.6% (Ministry of Health, Labour and Welfare, n.d.). Aging causes declines in physical and oral functions. A representative manifestation of decreased physical function is sarcopenia. Sarcopenia also affects oral muscles, including those involved in swallowing. Dysphagia due to sarcopenia of general skeletal muscles and swallowing muscles is called sarcopenic dysphagia (Kuroda & Kuroda, 2012). Sarcopenia has been identified as an independent risk factor for dysphagia in community‐dwelling older people (Cha et al., 2019). Dysphagia is closely associated with oral function as well as pharyngeal, laryngeal, and respiratory functions. A concept associated with sarcopenia is frailty. Similar to sarcopenia, frailty is associated with oral function in community‐dwelling older people; a decrease in occlusal force, thicker masseter muscle, and low oral diadochokinesis (ODK) rate have been reported in community‐dwelling older people with frailty (Watanabe et al., 2017). The ODK test measures lip and tongue dexterity by pronunciation, and is widely used to measure oral and swallowing functions. Decreased oral and swallowing functions have been implicated in the development of aspiration pneumonia, which may be life‐threatening (van der Maarel‐Wierink et al., 2011). Therefore, the maintenance of and improvements in oral and swallowing functions in community‐dwelling older people are important for preventing aspiration pneumonia.

In community‐dwelling older people, frailty due to declines in physical function, mental function, and social interactions increases the difficulty of going outside and narrows the scope of the activities of daily living, which is also referred to as life‐space mobility. Previous studies reported a relationship between frailty and life‐space mobility in community‐dwelling older people; those with frailty had lower life‐space mobility in their daily lives (Portegijs et al., 2016) and were predisposed to frailty due to reduced activity (Xue et al., 2008). Based on these findings, older people with low life‐space mobility in daily life may have poor oral function.

As decreased social interaction also causes frailty, we need to focus on social networks in order to maintain oral function. Social networks are associated with oral health, such as the number of remaining teeth, dental caries, and periodontitis, and have a positive impact on behaviors that promote health, including oral health (Merchant et al., 2003; Tsakos et al., 2013). Nagayoshi et al. (2017) previously examined the relationship between social relationships and oral function and found that residents aged 40 years and older who frequently interacted with neighbors and participated in leisure activities had higher tongue pressure. To date, the relationship between oral function and life‐space mobility or social networks has not yet been directly examined. Therefore, to improve oral and swallowing functions and, thus, prevent aspiration pneumonia in community‐dwelling older people, physical and social life guidance needs to be provided with a focus on the activities of daily living and social networks in addition to training that directly improves oral function as well as oral health guidance.

We hypothesized that older people with high life‐space mobility, abundant social networks, and frequent interpersonal interactions have higher tongue pressure, ODK scores, lip pressure, and screening test score for dysphagia. The objective of the present study was to clarify the relationship between oral function and life‐space mobility or social networks in order to explore approaches that prevent declines in oral function.

## 2 METHODS

### 2.1 Participants

The study participants were older people who the participants of preventive long‐term care projects aimed at the maintenance or improvement of physical and mental functions between September 2016 and March 2017 in Asakuchi City, Japan. People with cerebrovascular disease, neuromuscular disease, dementia, hearing impairment, visual impairment, or those who had difficulty in understanding the study content were excluded. Public health nurses in Asakuchi city verbally asked the participants of preventive long‐term care projects to enroll in this study, and those who provided consent were included. We calculated 89 participants to be the sample size required for linear multiple regression using G*power (version 3.1.9.6, Heinrich‐Heine‐Universität Düsseldorf, Germany) with a statistical power of 95%, significance level of 5%, six predictors, and an effect size (*f* ^2^) of 0.15. There were 125 participants (31 men and 94 women) in preventive long‐term care projects during the period under study, with a final total of 113 (31 men and 82 women) included in our study. The reason for dropout was that measurement of tongue pressure was unable to be performed due to the dental condition. All prospective participants were provided with both oral and written explanations about the purpose and content of the study, how the results would be published, and the protection of privacy, and written consent was obtained for study participation. The present study was conducted after approval by the ethics review committee of Kibi International University (No. 15‐02).

### 2.2 Assessment of life‐space mobility

Life‐space mobility was assessed using the life‐space assessment (LSA) questionnaire. Answers were filled in by the participants themselves or obtained through verbal communication. LSA involves the assessment of activity based on the frequency of activity at five spatial levels (bedroom/sleeping area, external area of the residence, neighborhood, inside the city, and outside the city). LSA scores were calculated based on these locations, the frequency of going out (less than once a week, 1–3 times a week, 4–6 times a week, or daily), the use of human assistance in going out, and the use of assistive devices in the 4 weeks prior to the assessment. Total scores obtained by summing the scores for each level ranged between 0 and 120 points. A score of 0 indicates that the area of activity is limited to the bedroom/sleeping area, while a score of 120 indicates that the participant frequently went out in his/her city of residence without assistance.

### 2.3 Assessment of social networks

Social networks were assessed using the Lubben Social Network Scale‐6 (LSNS‐6) questionnaire, with answers being filled in by the participants themselves or obtained through verbal communication. LSNS‐6 comprises six items that examine the number of relatives, friends, and neighbors with whom the individual interacts with in various daily situations, and the range of total scores obtained by summing the scores for each item is between 0 and 30 points. A higher score indicates a more abundant social network, with a score of 0 indicating that the individual interacts with no one on a daily basis.

### 2.4 Assessment of oral function

Oral function was assessed using four test items. Maximum voluntary tongue pressure was measured using a hand‐held balloon probe and manometer (JM‐TPM02, JMS Co., Ltd., Hiroshima, Japan). Participants were seated in a chair and a balloon probe was held in front of the palate. The jaw was then closed and the balloon probe was pressed with the tongue toward the palate with maximum voluntary effort for 7 s. This was repeated three times at intervals of 30 s, and the average of these measurements was adopted as the maximum voluntary tongue pressure.

Repetitive saliva swallowing test (RSST) is a widely used screening test for dysphagia in Japan. Participants sat in a chair and swallowed saliva or air voluntarily and repeatedly as fast as possible in 30 s. The middle finger of the examiner was placed just above the laryngeal prominence, and the index finger was placed further distal to the middle finger. The number of times the laryngeal prominence was elevated beyond the two fingers in 30 s was counted by palpation.

In the ODK test, we examined how many times participants repeated the “pa” syllable in 5 s in a sitting position. The number of articulations per 5 s was measured using a digital counter (Kenko‐Kun Handy, T.K.K. 3351, Takei Scientific Instruments Co., Ltd., Niigata, Japan), and the average number of articulations per second was calculated. The number of articulations per second was also calculated for the “ta” and “ka” syllables using the same procedure.

Maximum voluntary lip pressure was measured using a button with a diameter of 3 cm and a spring scale (Liter Meter, Oral Academy Co., Ltd., Tokyo, Japan). Participants sat in a chair and held a button with a thread between the lip and incisors. The examiner then pulled the spring balance connected to the thread passing through the button hole, and participants closed their lips with maximum effort to prevent the button from coming off. This was performed twice at an interval of 30 s, and the average of two measurements was adopted as the lip closure force.

One examiner performed the assessments other than the ODK test after calibration of the equipment. Two examiners carried out the ODK test after calibration of equipment.

### 2.5 Assessment of frailty

Frailty was assessed using grip strength and the Kihon Checklist.

Fried et al. (2001) defined physical frailty based on weight loss, subjective fatigue, a decline in the activities of daily living, reduced physical ability (gait speed), and decreased muscle strength (grip strength). As such, we employed grip strength. A strong correlation has been reported between grip strength and the total mass of skeletal muscles in older people (Yamada et al., 2013). Grip strength was measured using a Smedley hand dynamometer (T.K.K. 5401 GRIP‐D digital, Takei Scientific Instruments Co., Ltd., Niigata, Japan) for both the dominant and non‐dominant hands. The larger of the two measurements was used.

The Kihon Checklist was created by the Japanese Ministry of Health, Labour and Welfare to identify older people requiring care and support, and it is useful for the screening of frailty (Satake et al., 2016). The Kihon checklist is a simple self‐reporting yes/no survey consisting of 25 questions regarding the instrumental (three questions) and social (four questions) activities of daily living, physical functions (five questions), the nutritional status (two questions), oral function (three questions), cognitive function (three questions), and depressive mood (five questions; Satake et al., 2016). In the Kihon Checklist, points are counted when the responder answers “No” to each question. The higher the total score, the greater the likelihood of having frailty. In our analysis, the total score of 14 questions on the instrumental and social activities of daily living, physical functions, and the nutritional status was used as the score for the frailty of physical function in order to avoid statistical multicollinearity with the objective oral function test performed in the present study and also to focus on the frailty of physical function. We calculated the sum of the scores for three questions on oral function to assess the proportion of participants who were subjectively aware of decreased oral function.

## 3 STATISTICAL ANALYSIS

Age and all measurement results from LSA, LSNS‐6, oral function items, grip strength, and the Kihon Checklist were compared between male and female using the Student's *t*‐test. We also performed a univariate correlation analysis using Pearson's product moment correlation coefficient to clarify relationships among the measurement results of LSA, LSNS‐6, oral function items, grip strength, and the Kihon Checklist. A multiple regression analysis was then performed to evaluate the effects of life‐space mobility and social networks on oral function. Oral function was reported to be related to frailty and age in community‐dwelling older people (Watanabe et al., 2017); however, it currently remains unclear whether life‐space mobility and social networks are associated with oral function independently of these factors. Therefore, a multiple regression analysis was performed using a stepwise method incorporating each oral function item as a dependent variable and sex, LSA, and LSNS‐6 as independent variables, while grip strength, age, and the Kihon Checklist score were forced into the model as confounding factors for adjustments. All statistical analyses were performed using IBM SPSS Statistics version 23 (IBM Corp., Armonk, NY). The significance level was set at 5% for all analyses.

## 4 RESULTS

### 4.1 Participant characteristics

The mean age of all participants was 75.7 ± 7.3 years, with a mean age of 75.3 ± 7.7 years for men and 75.9 ± 7.1 years for women. The results of the Kihon Checklist showed that 63 participants (56%) were subjectively and at least slightly aware of a decline in oral function. Results obtained on age, body mass index (BMI), LSA, LSNS‐6, oral function, and frailty are shown in Table 1. The only significant difference observed between men and women was grip strength, which was significantly higher in men. The grip strength was 32.2 ± 6.7 kg for men and 21.8 ± 4.8 kg for women.

**TABLE 1:** Participant characteristics

	Total	Male	Female	*p* Value
*N*	113	31	82	
Mean age (years)	75.7 ± 7.3	75.3 ± 7.7	75.9 ± 7.1	.70
Mean Body Mass Index (kg/m^2^)	22.4 ± 3.2	22.0 ± 3.2	22.6 ± 3.2	.38
LSA	88.0 ± 22.4	88.4 ± 25.3	87.9 ± 21.4	.92
LSNS‐6	15.9 ± 5.6	16.7 ± 5.8	15.6 ± 5.5	.36
Oral function				
Tongue pressure (kPa)	29.3 ± 8.3	31.0 ± 10.2	28.6 ± 7.4	.18
RSST(times/30 s)	4.7 ± 2.3	5.1 ± 2.7	4.5 ± 2.2	.22
ODK “pa” (times/s)	5.8 ± 0.9	5.9 ± 1.0	5.8 ± 0.9	.58
ODK “ta” (times/s)	5.7 ± 1.0	5.7 ± 1.3	5.8 ± 0.8	.74
ODK “ka” (times/s)	5.4 ± 1.2	5.1 ± 1.5	5.6 ± 1.1	.08
Lip pressure(kg)	1.0 ± 0.4	1.1 ± 0.4	0.9 ± 0.4	.13
Frailty				
Grip strengh (kg)	24.7 ± 7.1	32.2 ± 6.7	21.8 ± 4.8	<.01
Kihon checlist score	2.4 ± 2.1	2.1 ± 2.0	2.5 ± 2.2	.43

*Note*: All data are shown as the mean ± *SD*.

Abbreviations: LSA, Life‐Space Assessment; LSNA‐6, Lubben Social Network Scale‐6; ODK, oral diadochokinesis; RSST, repetitive saliva swallowing test.

### 4.2 Bivariate simple correlation analysis of each parameter

The results of the correlation analysis are shown in Table 2. Age was correlated with the Kihon Checklist score (*r* = .38, *p* < .01), grip strength (*r* = −.38, *p* < .01), lip pressure (*r* = −.26, *p* < .01), ODK score for the “ta” syllable (*r* = −.29, *p* < .01), tongue pressure (−.27, *p* < .01), and LSA (*r* = −.26, *p* < .01). The LSA was correlated with tongue pressure (*r* = .67, *p* < .01), grip strength (*r* = .19, *p* < .05), ODK scores for the “pa” syllable (*r* = .26, *p* < .01), “ta” syllable (*r* = .24, *p* < .01), and “ka” syllable (*r* = .20, *p* < .05), LSNS‐6 (*r* = .22, *p* < .05), and Kihon Checklist score (*r* = −.29, *p* < .01). Grip strength was correlated with the Kihon Checklist score (*r* = −.29, *p* < .01).

**TABLE 2:** Bivariate simple correlation analysis of each parameter

	Kihon checlist score	Grip strengh	Lip pressure	ODK “ka”	ODK “ta”	ODK “pa”	RSST	Tongue pressure	LSNS‐6	LSA	Age
Age	0.38^**^	−0.38^**^	−0.26^**^	−0.14	−0.29^**^	−0.18	−0.01	−0.27^**^	−0.10	−0.26^**^	1
LSA	−0.29^**^	0.19^*^	0.11	0.20^*^	0.24^**^	0.26^**^	0.22^*^	0.67^**^	0.22^*^	1	
LSNS‐6	−0.14	0.13	0.02	−0.08	0.04	0.04	−0.01	0.12	1		
Tongue pressure	−0.31^**^	0.36^**^	0.15	0.20^*^	0.30^**^	0.25^**^	0.18	1			
RSST	−0.02	0.21^*^	0.04	0.06	0.14	0.26^**^	1				
ODK “pa”	−0.18	0.24^*^	0.10	0.45^**^	0.63^**^	1					
ODK “ta”	−0.25^**^	0.30^**^	0.19^*^	0.57^**^	1						
ODK “ka”	−0.06	0.01	0.00	1							
Lip pressure	−0.20^*^	0.28^**^	1								
Grip strengh	−0.29^**^	1									
Kihon checlist score	1										

Abbreviations: LSA, Life‐Space Assessment; LSNA‐6, Lubben Social Network Scale‐6; ODK, oral diadochokinesis; RSST, repetitive saliva swallowing test.

^*^ *p* < .05;

^**^ *p* < .01.

### 4.3 Association of life space‐mobility and social networks with oral function

Multiple regression analysis was performed to assess whether life‐space mobility and social networks are associated with each oral function item independently of frailty. Age, LSA, LSNS‐6, grip strength, age, and the Kihon Checklist score were incorporated as independent variables in the multiple regression analysis. A predictive model for the association of tongue pressure with the other variables is shown in Table 3. LSA (*B* = 0.222, *p* < .001), and grip strength (*B* = 0.266, *p* = .003) was associated with tongue pressure. The goodness of fit of the predictive model was an adjusted *R* ^2^ value of .486. A predictive model for the association of RSST scores with the other variables is shown in Table 4. The LSA (*B* = 0.023, *p* = .023) and grip strength (*B* = 0.074, *p* = .027) were associated with RSST scores. The goodness of fit of the predictive model was an adjusted *R* ^2^ of .060. A predictive model for the association of the ODK score for the “pa” syllable with the other variables is shown in Table 5. The LSA was associated with the ODK score for the “pa” syllable (*B* = 0.008, *p* < .041). The goodness of fit of the predictive model was an adjusted *R* ^2^ of .078. Predictive models for the association of the ODK score for the “ta” and “ka” syllables, and lip pressure with the other variables are shown in Tables 6, 7, and 8, respectively. No variables were associated with ODK scores for the “ta” and “ka” syllables or lip pressure.

**TABLE 3:** Multiple regression analysis for tongue pressure

	*β*	*B*	95% confidence interval for B	*p* Value	*R* ^2^	Adjusted *R* ^2^
					.504	.486
LSA	0.602	0.222	0.170 to 0.275	<.001		
Grip strength	0.229	0.266	0.094 to 0.438	.003		
Age	−0.006	−0.006	−0.182 to 0.169	.942		
Kihon checlist score	−0.062	−0.242	−0.826 to 0.342	.414		

Abbreviation: LSA, Life‐Space Assessment.

**TABLE 4:** Multiple regression analysis for RSST

	*β*	*B*	95% confidence interval for *B*	*p* Value	*R* ^2^	Adjusted *R* ^2^
					.094	.060
LSA	0.225	0.023	0.003 to 0.044	.023		
Grip strength	0.226	0.074	0.008 to 0.140	.027		
Age	0.105	0.034	−0.033 to 0.101	.322		
Kihon checlist score	0.071	0.078	−0.146 to 0.302	.490		

Abbreviation: LSA, Life‐Space Assessment.

**TABLE 5:** Multiple regression analysis for ODK “pa”

	*β*	*B*	95% confidence interval for *B*	*p* Value	*R* ^2^	Adjusted *R* ^2^
					.111	.078
LSA	0.200	0.008	0.000 to 0.017	.041		
Grip strength	0.170	0.023	−0.004 to 0.049	.092		
Age	−0.035	−0.005	−0.031 to 0.022	.735		
Kihon checlist score	−0.063	−0.028	−0.118 to 0.061	.533		

Abbreviations: LSA, Life‐Space Assessment; ODK, oral diadochokinesis.

**TABLE 6:** Multiple regression analysis for ODK “ta”

	*β*	*B*	95% confidence interval for *B*	*p* Value	*R* ^2^	Adjusted *R* ^2^
					.157	.126
LSA	0.142	0.006	−0.002 to 0.014	.133		
Grip strength	0.187	0.025	−0.001 to 0.052	.058		
Age	−0.149	−0.020	−0.047 to 0.007	.144		
Kihon checlist score	−0.094	−0.043	−0.132 to 0.046	.345		

Abbreviations: LSA, Life‐Space Assessment; ODK, oral diadochokinesis.

**TABLE 7:** Multiple regression analysis for ODK “ka”

	*β*	*B*	95% confidence interval for *B*	*p* Value	*R* ^2^	Adjusted *R* ^2^
					.051	.016
LSA	0.183	0.01	−0.001 to 0.020	.069		
Grip strength	−0.064	−0.011	−0.045 to 0.024	.534		
Age	−0.122	−0.02	−0.055 to 0.015	.261		
Kihon checlist score	0.017	0.01	−0.107 to 0.127	.868		

Abbreviations: LSA, Life‐Space Assessment; ODK, oral diadochokinesis.

**TABLE 8:** Multiple regression analysis for lip pressure

	*β*	*B*	95% confidence interval for *B*	*p* Value	*R* ^2^	Adjusted *R* ^2^
					.113	.089
Grip strength	0.195	0.011	0.000 to 0.022	.051		
Age	−0.155	−0.009	−0.020 to 0.003	.134		
Kihon checlist score	−0.088	−0.017	−0.054 to 0.020	.373		

## 5 DISCUSSION

The present study included community‐dwelling older people with ages ranging between 55 and 89 years. Referring to the definition of oral frailty reported by Tanaka et al. (2018), the average tongue pressure and ODK score of participants did not fit the definition of oral frailty. The condition of teeth or masticatory function was not examined in the present study. Nevertheless, many participants presumably had oral pre‐frailty or oral frailty because 56% were subjectively aware of decreased oral function.

Previous studies reported that age (Utanohara et al., 2008; Youmans & Stierwalt, 2006), grip strength (Buehring et al., 2013; Butler et al., 2011), and jump height (Buehring et al., 2013) were associated with maximum voluntary tongue pressure. These studies suggested that a decrease in skeletal muscle mass and muscle strength due to sarcopenia is associated with the maximum voluntary tongue pressure. In our multiple regression analysis, grip strength and life‐space mobility were adopted as independent factors associated with tongue pressure, whereas age was not. The results obtained in the present study may be attributed to only a small proportion of participants having physical frailty or sarcopenia; however, a relationship exists between generalized skeletal muscles and grip strength even if sarcopenia does not appear to be present. In contrast, the Kihon Checklist score had no association, possibly because the average score was low in the present study due to the small number of participants with physical frailty. Regarding the association of tongue pressure with life‐space mobility, participants with a wider range of activities were presumed to be more active, did not have sarcopenia or frailty, and had a higher general muscle strength. LSA examines the range of activity; however, the amount of physical activity was not assessed in detail in the present study. Tsai et al. (2015) previously reported a relationship between life‐space mobility and physical activity, with a higher LSA score indicating more physical activity.

RSST assesses whether the swallowing reflex is sufficiently elicited and is affected by the amount of saliva produced (Oguchi et al., 2000). Physiological changes induced by presbyphagia include difficulties with the initiation of swallowing due to reduced olfactory and gustatory sensitivities (Nogueira & Reis, 2013), which may also affect RSST. In the present study, grip strength was adopted as an independent factor associated with RSST in the multiple regression analysis; however, as the goodness of fit was poor, it was not judged to be a direct association. Potential reductions in olfactory and gustatory sensitivities, and saliva volume were not examined; therefore, we assumed factors other than those measured to be associated with RSST and grip strength to not be the only interacting factor.

The measurement of ODK is primarily based on the dexterity of the lips and tongue. Izuno et al. (2016) found a relationship between the dexterity and agility of the lips and tongue and grip strength, in addition to general physical performance, suggesting that the general muscle condition, including occlusal condition, physical weakness, and sarcopenia, is associated with physical performance and perioral muscle activity. Watanabe et al. (2017) also showed that a population with physical frailty had lower occlusal force, a thinner masseter muscle, and lower ODK rates than “robust” people. A path analysis conducted in a previous study revealed that tongue pressure affected the ODK rate (Kugimiya et al., 2019). In the multiple regression analysis, the LSA was used as an independent factor associated with the ODK score for the “pa” syllable; however, as the goodness of fit was poor, it was not judged to be a direct association. No variables were adopted as factors correlating with ODK scores for the “ta” and “ka” syllables and lip pressure. These results suggest that muscle power factors, such as tongue pressure, are related to overall skeletal muscle mass and strength, whereas muscle control factors, such as ODK, are not.

The LSNS‐6, which is employed to assess social networks, was not adopted as a factor associated with any oral function in the multiple regression analysis. Nagayoshi et al. (2017) speculated that living with a family and having good neighbors may increase the opportunity to mingle with others, thereby increasing the frequencies of eating, talking, and laughing together. They also suggested that increased muscle activity around the mouth and pharynx is beneficial for tongue function. Based on this study, we hypothesized that the LSNS‐6 can be adopted as a factor associated with oral function, including tongue pressure. However, it was not adopted for this purpose in the present study for two reasons. The first reason is that LSNS‐6 evaluates the number of people with whom one interacts with on a daily basis, but not the frequency of interactions. Therefore, even if one interacts with a large number of people, he/she may be regarded as spending less time using muscles around the mouth and pharynx if he/she spends only a short time with his/her relative or friend. There was no association between oral function and LSNS‐6 because it is difficult to conclude that LSNS‐6 accurately assesses the frequencies of oral and pharyngeal activities. In the univariate analysis, a weak correlation was observed between LSA and LSNS‐6, suggesting that many participants with high life‐space mobility frequently interacted with others on a daily basis. The second reason is the importance of focusing on tongue activity in daily life to elucidate the mechanisms by which tongue pressure is increased. Searl and Evitts (2013) measured the articulatory contact pressure (ACP) between the tongue and palate during casual conversations and the formation of clearer sounds in speech (i.e., articulation) in healthy subjects. They reported that ACP was the highest for the “t” phoneme (4.49 ± 2.24 kPa) and the lowest for the “l” phoneme (1.78 ± 1.05 kPa) during conversations. The magnitude of tongue pressure resistance during tongue pressure resistance training was previously reported to be “as forcefully as possible” (Namiki et al., 2019; Wakabayashi et al., 2018) and between 60 and 80% of maximum tongue pressure (Robbins et al., 2005; Yano et al., 2019). In muscle strengthening training for generalized skeletal muscles, resistance exercise of low intensity with isometric contractions at 30% of the maximal voluntary contraction was found to increase muscle tissue after 16 weeks (Always et al., 1990). With resistance exercise at less than 30% of the maximal voluntary contraction, only increases in physical endurance were reported (Mettler & Griffin, 2016). Even with the tongue pressure cut‐off value for oral frailty, difficulties are associated with sustaining ACP for phonation in daily life at a force of at least 30% of the maximum voluntary tongue pressure, and this pressure is insufficient to increase tongue pressure. Thus, we consider it impossible to maintain tongue pressure through social interactions alone.

The present study suggests that older people with high tongue pressure are able to maintain their overall skeletal muscle mass and strength due to the wide range of activities of daily life. Miyazaki and Mori (2020) previously reported that karaoke training contributed to higher tongue pressure and better respiratory function. Searl and Evitts (2013) showed that ACP was markedly higher during articulation than during conversations. Accordingly, oral function training using phonation in combination with physical activity may be effective. Although no direct associations with social networks were found in the present study, we consider social networks to also be an important factor that indirectly lead to life‐space mobility because having more relatives and friends increases motivation to go outside. The factors associated with the ODK and lip pressure, which are indices of the dexterity of the lips and tongue, were not fully elucidated in this study. Therefore, further studies are needed to clarify which oral function is related to speech frequency by establishing a method for objectively evaluating speech frequency.

## 6 CONCLUSION

Life‐space mobility and grip strength were independent factors associated with the maximum voluntary tongue pressure, whereas social networks were not directly associated with oral function. Regarding tongue pressure, the prevention and attenuation of sarcopenia through increased physical activity are important. Collectively, the present study suggests the necessity of a program that approaches both oral and physical functions through guidance for securing a certain amount of daily activity, and preventive long‐term care projects for older people at risk of or exhibiting a decline in maximum voluntary tongue pressure. As this was a small cross‐sectional study, a large longitudinal study is required in the future to clarify this.

## CONFLICT OF INTEREST

The authors declare thre is no conflict of interest.

## Data Availability

The data that support the findings of this study are available on request from the corresponding author. The data are not publicly available due to privacy or ethical restrictions.
